# Bridging therapeutic opportunities: a survey by the Italian molecular tumor board workgroup of Alliance Against Cancer

**DOI:** 10.1186/s13046-022-02512-0

**Published:** 2022-10-17

**Authors:** Gennaro Ciliberto, Marco Canfora, Irene Terrenato, Chiara Agnoletto, Francesco Agustoni, Loredana Amoroso, Gustavo Baldassarre, Giuseppe Curigliano, Angelo Delmonte, Antonella De Luca, Michelangelo Fiorentino, Vanesa Gregorc, Toni Ibrahim, Chiara Lazzari, Angela Mastronuzzi, Paolo Pronzato, Armando Santoro, Giovanni Scambia, Stefania Tommasi, Andrea Vingiani, Patrizio Giacomini, Ruggero De Maria

**Affiliations:** 1 IRCCS Istituto Nazionale Tumori Regina Elena, Rome, Italy; 2 ROV, Istituto Oncologico Veneto IOV-IRCCS, Padua, Italy; 3 Fondazione IRCCS Policlinico San Matteo, Pavia, Italy; 4 IRCCS Istituto Giannina Gaslini, Genoa, Italy; 5 Centro Di Riferimento Oncologico Di Aviano IRCCS, Aviano, Italy; 6 Istituto Europeo Di Oncologia IRCCS, Milan, Italy; 7 Dipartimento Di Oncologia Ed Emato-Oncologia, Università La Statale Di Milano, Milan, Italy; 8 Istituto Romagnolo Per Lo Studio Dei Tumori “Dino Amadori” - IRST IRCCS, Meldola, Italy; 9 Istituto Nazionale Tumori-IRCCS-Fondazione G. Pascale, Naples, Italy; 10 IRCCS Policlinico Sant’ Orsola-Malpighi, Bologna, Italy; 11 Istituto Di Candiolo - FPO (Fondazione del Piemonte Per L’Oncologia) IRCCS, Candiolo, Italy; 12 IRCCS Istituto Ortopedico Rizzoli, Bologna, Italy; 13 IRCCS Ospedale San Raffaele, Milan, Italy; 14 IRCCS Ospedale Pediatrico Bambino Gesù, Rome, Italy; 15 IRCCS Humanitas Research Hospital-Humanitas Cancer Center, Rozzano, Milan Italy; 16 Department of Biomedical Sciences, Humanitas University, Pieve Emanuele, Milan, Italy; 17 Dipartimento Di Ostetricia E Ginecologia, Università Cattolica del Sacro Cuore, Rome, Italy; 18 Fondazione Policlinico Universitario A. Gemelli IRCCS, Rome, Italy; 19 IRCCS Istituto Tumori “Giovanni Paolo II”, Bari, Italy; 20 Fondazione IRCCS Istituto Nazionale Dei Tumori, Milan, Italy; 21 Dipartimento Di Medicina E Chirurgia Traslazionale, Università Cattolica del Sacro Cuore, Rome, Italy

**Keywords:** Molecular tumor boards, MTB, Molecular alterations, Targeted anticancer drugs, Alliance against Cancer

## Abstract

**Background:**

Molecular tumor boards (MTBs) match molecular alterations with targeted anticancer drugs upon failure of the available therapeutic options. Special and local needs are most likely to emerge through the comparative analysis of MTB networks, but these are rarely reported. This manuscript summarizes the state-of-art of 16 active Italian MTBs, as it emerges from an online survey curated by Alliance Against Cancer (ACC).

**Main text:**

Most MTBs (13/16) are exclusively supported through local Institutional grants and meet regularly. All but one adopts a fully virtual or a mixed face-to-face/virtual calling/attendance meeting model. It appears that the ACC MTB initiative is shaping a hub-and-spoke virtual MTB network reminiscent of non-redundant, cost-effective healthcare organization models. Unfortunately, public awareness of MTB opportunities presently remains insufficient. Only one center has a website. Dedicated e-mail addresses are for the exclusive use of the MTB staff. More than half of ACC members consider a miscellanea of most or all solid and hematological malignancies, and more than one-third consider neoplasms arising at any anatomical location. The average number of Staff Members in MTBs is 9. More than 10 staff members simultaneously attend MTB meetings in 13 MTBs. A medical oncologist is invariably present and is in charge of introducing the clinical case either with (45%) or without previous discussion in organ-specific multidisciplinary Boards. All but two MTBs take charge of not only patients with no standard-of-care (SoC) therapy option, but also cases receiving NGS profiling in SoC settings, implying a larger number of yearly cases. All MTBs run targeted NGS panels. Three run whole-exome and/or RNAseq approaches. ESCAT-ESMO and/or Onco-KB levels of evidence are similarly used for diagnostic reporting. Most MTBs (11) provide a written diagnostic report within 15 days. Conclusions are invariably communicated to the patient by the medical oncologist.

**Conclusions:**

MTB networking is crucial not only for molecular diagnosis and therapy assignment, but also for healthcare governance. Survey results show that MTBs review therapeutic opportunities at the crossover between standard-of-care with off-label, the former task being much beyond their scope. Societal and scientific implications of this beyond-the-scope MTB function may be relevant for healthcare in Italy and abroad.

**Supplementary Information:**

The online version contains supplementary material available at 10.1186/s13046-022-02512-0.

## Background

Molecular tumor boards (MTBs) match molecular alterations with targeted anticancer drugs upon failure of the available therapeutic options [1]. Straightforward in principle, this approach is complicated due to the considerable combinatorial variety of tumors and drugs. To assemble the diverse, necessary pieces of expertise, and to harmonize potentially divergent MTB layouts, MTBs worldwide have undergone a transition toward virtual formats (vMTBs). Some interesting examples are: the *e-Tumor Boards* of the Memorial Sloan–Kettering Cancer Center, the University of Pittsburgh MTB [2], the liquid biopsy-oriented *OncoSET* Northwestern University (in the in USA), the *EXPeRT* pediatric advice group in Dortmund, the MITO gynecology network [3], the *Heidelberg NCT* tumor board, and the *Hamburg UKE group* (in the EU), just to mention a few. Key to virtualization are shared multi-center decision support tools, such as the MTB Precision Oncology Portal of Cancer Core of Europe [4], the Precision Medicine MTB platform [5], and the *GeNeo and BALLETT* umbrella-core-initiatives of the Belgian Society for Medical Oncology. Additional vMTB/network study formats have been reviewed [6]. In such a complex landscape, opinion polls and surveys may help to highlight special and local needs within and across networks, and to empower best practices, but to our knowledge they have rarely [7] been reported.

Alliance Against Cancer (*ACC*) is the largest Italian network of cancer research and clinical institutes [5]. It currently includes 26 research hospitals and a patient association under the high patronage of the Italian Ministry of Health. In 2020, the ACC MTB Workgroup collegially defined patient eligibility criteria, minimal technical requirements for genomic testing, a common ethical framework, a General Data Protection Regulation-compliant code of conduct for the processing and management of personal data, and a capacity-building/educational plan linking the MTBs within the ACC network with higher education training programs (e.g., university master degrees and the continuous medical education system). Along with the *Italian Association for Medical Oncology recommendations*, *ACC MTB guidelines* represent one of the first attempts in Italy to harmonize procedures and set the stage for equal MTB access.

Although full guideline compliance is not mandatory for ACC member institutions, recommendations resulted from a unanimous consensus. Therefore, it was of interest to monitor their application against a background of diverse local conditions and to assess the overall progress and achievements of our multi-center MTB effort in Italy during the years 2020 and 2021. On this basis, a multiple-choice questionnaire was designed by Research Electronic Data Capture (REDCap) and posted online in two rounds (41 and 7 questions, respectively, listed in Additional file 1: Appendix I). The latter round, inspired by peer review, was only for ACC Members with an officially endorsed MTB. Embedded Form Display Logic generated a hierarchical decision tree conditionally routing respondents to mutually exclusive sets of menus with internal self-consistency checks. The questionnaires (elaborated by IBM-SPSS v.21.0) gathered information on 5 distinct topics, each of which is the subject of a separate paragraph below: (1) legal issues and budget; (2) tumors profiled; (3) MTB organization and case-mix; (4) next-generation sequencing (NGS) technical tools; and (5) diagnostic reporting.

## Main text

Responses were recorded by all the 26 ACC research hospitals: although 19/26 MTBs declared an active (or being activated) MTB, only 11 had received an Institutional (legally binding) endorsement, and only 3 were able to obtain official recognition by the regional health authorities (the Italian Healthcare System is federal). Most ACC MTBs (13/16 respondents) are exclusively supported through local Institutional grants by university departments and the Direction Offices of the National Cancer Institutes. Support from the National Health System (either direct or through regional authorities) is declared in 3 cases only. Three MTBs could obtain donations from pharmaceutical companies, but none covers drug supply. Altogether, 8/18 (45%) responding centers declared funding from one source; 6 (33%) centers from more than one source and 4 (22%) centers were unable to secure a budget under any form.

Despite limitations, most MTBs meet regularly: monthly (1/16 respondents, 6%), every 15 days (5/16, 31%), or weekly (4/16; 25%). Only a minority convene 'when needed' (25%, *n* = 4) or have not yet defined a schedule (2/16, 13%). Interestingly, all but one ACC member adopt a fully virtual (10/17) or a mixed (virtual plus face-to-face; 6/17) calling/attendance meeting model. Accordingly, in-house information technology (IT) platforms and web apps were adopted/customized by most ACC members for internal management, data recording/annotation, query, and analysis. Although somewhat heterogeneous, this embryonal IT system has prepared ACC to take on future interoperability challenges [4, 8]. Along this line, 14/16 (88%) active MTBs accept not only inpatients but also outpatients from outside their Institutions.

It then appears that the ACC MTB initiative is shaping a hub-and-spoke virtual MTB network reminiscent of non-redundant, cost-effective healthcare organization models. Unfortunately, public awareness of MTB opportunities presently remains insufficient. Only one center has a website, whereas dedicated email addresses (available in 9/16; 56% of the cases) are for the exclusive use of the MTB staff. The Italian Ministry of Health has recently outlined an overall strategy envisaging the creation of MTB Regional networks operating under the guidance of the Agency for the development of the Health System (AGENAS). However, specific guidance for implementing this regionally centered model has not yet been disclosed.

**Fig. 1:**
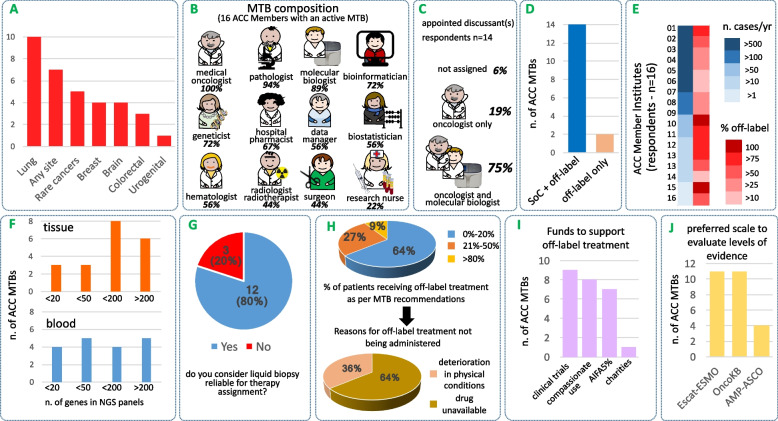
The ACC MTB network. **A** Most frequently discussed neoplasms. Respondents were given the option to tick more than one in a series of multiple choices. **B** MTB staff members ranked by frequency. **C** Frequently appointed discussants. **D** Case-mix: MTBs assigning treatment both in indication (SoC) and off-label (blue), and off-label only (orange). **E** numerosity and case-mix: MTBs (n. 1–16) were ranked by the number of cases discussed per year (descending order; shades of blue). Each MTB is also pseudo-colored in shades of red (color intensity proportional to case-mix as defined in D). **F** Confidence in liquid biopsy. **G** Complexity (n. of genes) of clinical NGS panels used for tissue and blood. **H** Failure to administer MTB-recommended off-label treatment and causes thereof. **I** Funds to support off-label treatment (**J**) Top three international scales of actionable biomarkers (levels of evidence) adopted by the ACC MTB Workgroup

More than half (10/18; 56%) of ACC members consider a miscellanea of most or all solid and hematological malignancies (including in some cases pediatric tumors), and more than one-third (7/18; 39%) consider neoplasms arising at any anatomical location (Fig. 1A). Lung tumors are most prevalent (10/18; 56%), but rare tumors are seen very frequently (5/18; 28%), e.g. MTBs in Italy focus on neoplasms that are either very rich or very poor in actionable markers. It will be of interest to determine whether a similar polarization occurs worldwide.

The average number of Staff Members in MTBs is 9, > 10 staff members simultaneously attending MTB meetings in 13/18 (72%) ACC MTBs. A medical oncologist is invariably present, followed in frequency by the other professionals listed in Fig. 1B. As expected, the oncologist is most often (11/16; 69%) in charge of introducing the clinical case either with (45%, *n* = 5) or without (*n* = 6, 55%) previous discussion in organ-specific multidisciplinary Boards (e.g., Disease Management Teams). Subsequently, an oncologist and a molecular biologist are jointly appointed for case updates in most (12/16; 75%) centers (Fig. 1C). Again, this highlights the MTB's quintessential mission: matching patients to actionable alterations.

An unexpected finding from our survey is that all but two MTBs (*n* = 14/16, 88%) take charge of not only patients with no standard-of-care (SoC) therapy option, but also cases receiving NGS profiling in SoC settings (Fig. 1D). A SoC/non-SoC case-mix implies a larger number of yearly cases (Fig. 1E, shades of blue) compared to the two non-SoC-only outliers (Fig. 1E, MTBs n. 10 and 15, shades of red). Further local differences in case-mix may arise due to one or more of the following: (a) no or insufficient previous genomic profiling; (b) request for an additional expert opinion; (c) need to locate a suitable clinical trial; (d) need to plan personalized treatments ahead of time; and (e) approval of the relevant biomarker by FDA and/or EMA but not by the National Regulatory Agency (Agenzia Italiana del Farmaco [AIFA]). Thus, although far beyond their primary scope, MTBs in Italy routinely review the evolving scenario of therapeutic opportunities. This unappreciated task deserves future surveys and harmonization.

All 16 active MTBs run targeted NGS panels, and three run whole-exome and/or RNAseq approaches. Gene panels for tissues and blood have similar complexity (Fig. 1F), and the latter are considered fully reliable to assign therapy by 12/15 respondents (Fig. 1E). This is remarkable since NGS is not yet EMA-approved for liquid biopsy. The size of targeted panels appears not to affect (7/11 respondents; 64%) diagnostic turnaround times or, when this happens, delay exceeds 15 days in one case only (9%). Likewise, only 1/4 centers running exome sequencing declare a delay > 15 days in diagnostic reporting (Additional file 1: Appendix I). Although all ACC members may occasionally outsource from commercial vendors, most NGS assays are carried out in-house on own equipment. Thus, clinical NGS is by no means a critical MTB bottleneck.

ESCAT-ESMO and/or Onco-KB levels of evidence are similarly used (12/18; 67%). The intuitive user interface of the latter (www.oncokb.org) possibly explains its success outside the USA. Most MTBs (11/15 respondents; 73%) provide a written diagnostic report within 15 days (Fig. 1H), either as a simple statement (actionable level and corresponding class of therapeutic agents; 5 MTBs) or as a more comprehensive recommendation, including case discussion (the remaining 6 cases). This divergence suggests that standard MTB clinical summary formats are needed to facilitate medical communication, database annotation, and knowledgebase searches.

When multiple actionable alterations are detected, most ACC MTBs prioritize by ad hoc literature search, with a clear trend to use target therapy before checkpoint blockade, and extreme caution in considering off-label combinations (Additional file 1: Appendix I). Conclusions are invariably communicated to the patient by the medical oncologist. NGS profiling is completely free of charge for all ACC patients. Since securing off-label treatment is known to be difficult [9], we specifically asked how many patients who receive a recommendation do undergo treatment (Fig. 1H). Disappointingly, 7/11 (64%) respondents declare that less than 20% of MTB patients can be treated off-label. Patient refusal or oncologist’s choice are never reported as significant issues (Additional file 1: Appendix I). Worsening of patient conditions is indicated by 3/11 (36%) of responders, but the leading cause of therapy not being administered is by far (7/11; 64%) that the selected drug is ‘not available’ to the MTB. This answer must be interpreted in light of the extensive leveraging, by ACC MTBs, of all the available ‘drug supply sources' presently available in Italy, e.g. patient referral to clinical trials, so-called special AIFA 5% funds, and compassionate use including direct purchase through the Hospital Pharmacist (Fig. 1I). This is clearly the single most critical area emerging from our survey.

## Conclusions

A snapshot from the ACC vantage viewpoint revealed quadruplication of MTBs in Italy (4 to 16) in less than 4 years (2018–2021), fully virtual operational modes, and full-fledged NGS facilities. ACC Members perceive federated MTBs as a National Health priority and plan to generate an IT network. This may assist local governance (regions) and the AIFA Observatory of Clinical Trials in monitoring prescription appropriateness, hence alleviating cost. However, slow clearance of legally binding steps and insufficient drug availability/budgeting schemes are major hurdles to the widespread adoption of a federated MTB model in Italy. Unless rapidly amended, the present situation may adversely impact equal access to MTB expertise in Italy.

For many years, new cancer treatments have been introduced in Italy through managed-entry, risk-sharing, and payment-by-result agreements with pharmaceutical companies [10]. Shared cost/variable cohort models, such as DRUP [11], would be a logical extension of standard negotiation. At the same time, the Italian MTB network could ideally provide overarching governance and links to international initiatives/regulatory bodies.

Finally, our survey challenges the widespread idea that MTBs focus on off-label treatment. We have captured a transition phase of precision oncology that requires critical evaluation, selection, and 'bridging' of treatment opportunities, e.g. SoC, emerging (clinical trials) and off-label. Societal and scientific implications of this beyond-the-scope MTB function may be relevant for healthcare in Italy and abroad.

## Supplementary Information

**Additional file 1: Appendix I.** 

## Data Availability

All data generated or analyzed in this study are included in this article and/or its figures. Further enquiries can be directed to the corresponding author.

## Abbreviations

AIFA: Agenzia Italiana del Farmaco
ACC: Alliance Against Cancer
MTB: Molecular tumor board
NGS: Next-generation sequencing
SoC: Standard of care
