# Supplementary material for: Bridging therapeutic opportunities: a survey by the Italian molecular tumor board workgroup of Alliance Against Cancer
Source: J Exp Clin Cancer Res. 2022 Oct 17;41:305. doi: 10.1186/s13046-022-02512-0 (PMC9575294; doi:10.1186/s13046-022-02512-0)
Supplement: Supplementary file 1 — Additional file 1: Appendix I. [file 13046_2022_2512_MOESM1_ESM.pdf]

ACC-MTB Network Survey

ACC-MTB Network Survey

This survey aims at capturing MTB activities within the Alliance Against Cancer (Alleanza Contro il Cancro – ACC) network. There are no ‘right’ or ‘wrong’ responses. Results will be anonymised before sharing. The identity of responders will be encrypted and by no means specific responses will be associated with the identity of the ACC respondent Member

ACC Member Institute

- ◯ Centro Di Riferimento Oncologico - CRO, IRCCS
- ◯ Istituto Nazionale Tumori Regina Elena - IRE, IRCCS
- ◯ Istituto Nazionale Dei Tumori, INT- Fondazione IRCCS
- ◯ Ospedale Policlinico San Martino IRCCS
- ◯ Istituto Nazionale Tumori IRCCS - Fondazione Giovanni Pascale
- ◯ Istituto Tumori Giovanni Paolo II Bari - IRCCS Associati Ordinari
- ◯ Istituto Superiore di Sanità - ISS
- ◯ Azienda Unità Sanitaria Locale- IRCCS - Reggio Emilia
- ◯ Istituto Nazionale Neurologico C. Besta - Fondazione IRCCS
- ◯ Istituto Giannina Gaslini - IRCCS
- ◯ Istituto Oncologico Veneto - IOV, IRCCS
- ◯ Istituti Clinici Scientifici Maugeri - IRCCS
- ◯ Centro Di Riferimento Oncologico Di Basilicata - CROB, IRCCS
- ◯ Istituto Clinico Humanitas - ICH, IRCCS
- ◯ Ospedale San Raffaele - OSR, IRCCS
- ◯ Fondazione Luigi Maria Monti - IDI - IRCCS
- ◯ Istituto Europeo Di Oncologia - IEO, IRCCS
- ◯ Istituti Ortopedici Rizzoli - IOR, IRCCS
- ◯ Istituto Romagnolo per lo Studio dei Tumori Dino Amadori - IRST-IRCCS
- ◯ Istituto di Candiolo, FPO - IRCCS
- ◯ Ospedale Casa Sollievo Della Sofferenza - IRCCS
- ◯ Ospedale Bambino Gesù - OPBG, IRCCS
- ◯ Istituto Di Ricerca Diagnostica E Nucleare - SDN, IRCCS
- ◯ IRCCS Saverio De Bellis
- ◯ Fondazione Policlinico Universitario Agostino Gemelli, Roma - IRCCS
- ◯ Fondazione IRCCS Policlinico San Matteo Pavia
- ◯ Istituto di ricerche farmacologiche Mario Negri
- ◯ IRCCS Ospedale Sacro Cuore - Don Calabria
- ◯ Azienda Ospedaliero-Universitaria di Bologna, Policlinico di Sant'Orsola
- ◯ AIMaC - Associazione Italiana Malati Di Cancro
- ◯ ISG - Italian Sarcoma Group, Bologna
- ◯ Fondazione CNAO

1 Does your Institution host an officially endorsed (internal recognition) MTB?

11	◯ YES	
7	◯ NO	
8	◯ being activated	

If your answer was ‘no’, the remaining questions are irrelevant and your survey is completed, thank you.

No.	Question	Count	Option
2	When was your MTB established (date of official endorsement or first meeting)?	0	◯ 2016 Semester 1
		0	◯ 2016 Semester 2
		0	◯ 2017 Semester 1
		0	◯ 2017 Semester 2
		1	◯ 2018 Semester 1
		2	◯ 2018 Semester 2
		1	◯ 2019 Q1
		0	◯ 2019 Q2
		0	◯ 2019 Q3
		0	◯ 2019 Q4
		3	◯ 2020 Q1
		1	◯ 2020 Q2
		2	◯ 2020 Q3
		0	◯ 2020 Q4
		1	◯ 2021 Q1
		1	◯ 2021 Q2
		2	◯ 2021 Q3
		2	◯ 2021 Q4
3	How is financial support secured? (you may thick more than one box)	3	☐ Region
		2	☐ Current research (Ministry of Health)
		1	☐ Finalized research (AIRC, etc)
		0	☐ Finalized research (EU)
		1	☐ Finalized research (Ministry of Health)
		6	☐ Intramural Funds (e.g. Scientific Direction)
		0	☐ Intramural Funds (e.g. Sanitary Direction)
		1	☐ Intramural Funds (e.g. General Direction)
		7	☐ Intramural Funds (e.g. Department)
		7	☐ Other (specify)
	Specify	2 Funded by Company<br>1 External funding<br>1 To be defined	2 No funding<br>1 No answer
4	Your MTB considers	10	◯ Non-hematological neoplasms (solid tumors)
		1	◯ Hematological disorders
		7	◯ Both
5	If your MTB considers both, what is the approximate case composition?	6	◯ >90% non hematological
		0	◯ >90% hematological
		1	◯ any percentage in between

6	Who are the Professionals in your MTB? (you may thick one or more boxes)	18<br>10<br>17<br>15<br>16<br>13<br>10<br>10<br>13<br>10<br>8<br>9<br>8<br>12<br>4<br>0<br>4<br>9<br>1<br>1<br>7	☐ Oncologist<br>☐ Hematologist<br>☐ Pathologist (Histopathologist)<br>☐ Molecular Pathologist<br>☐ Molecular Biologist/Biotechnologist<br>☐ Bioinformatician<br>☐ Data manager<br>☐ Biostatistician<br>☐ Medical Geneticist<br>☐ Biologist with a degree in Genetics<br>☐ Radiologist<br>☐ Radiotherapist<br>☐ Surgeon<br>☐ Hospital Pharmacist/expert in Pharmacoeconomics<br>☐ Expert in Pharmacogenomics<br>☐ Patient Advocacy Delegate<br>☐ Nurse/Technician<br>☐ Scientific Secretary<br>☐ Administrative Secretary<br>☐ Other Administrative Personnel<br>☐ Other (specify)
	Role	1 Psycologist<br>1 Case Manager<br>1 Research staff<br>1 Virologist and Pediatrician	1 Clinical Trial Expert, Endocrinologist and Neurologist<br>1 Oncology Resident<br>1 To be defined
8	Do all professionals invariably convene or some MTB members are requested to attend only in specific MTB sessions?	6<br>11	◯ All MTB members are requested to always attend<br>◯ It depends on the clinical cases begin considered
9	Your MTB was established through:	12<br>1<br>2	◯ Institutional endorsement<br>◯ Regional (wider city area) endorsement<br>◯ Spontaneous MTB, no official endorsement
10	Also in light of the COVID 19 pandemics, does your MTB convene Face to Face (FtF), or online (virtual MTB, vMTB, any Telco platform)	1<br>10<br>6	◯ FtF only<br>◯ vMTB<br>◯ both
11	How often does your MTB convene?	4<br>5<br>1<br>6	◯ Weekly<br>◯ Every 15 days<br>◯ Monthly<br>◯ Other, specify
	Other	1 It depends on the number of cases to be discussed<br>2 To be defined<br>3 As needed	

12	Does your MTB consider inpatients only, or also patients referred from other institutions?	2<br>14	◯ Only inpatients<br>◯ Also outpatients
13	At which time is the expert MTB opinion requested?	14<br><br><br>2<br>0	◯ Metastatic cancer requiring genomic profiling including tumors for which genomic profiling is in indication<br>◯ Tumors with no standard therapeutic options<br>◯ Other (specify)
	Other		__________________________
14	How many cases per year (on average) are discussed at your MTB?	2<br>4<br>2<br>2<br>6	◯ 1-10<br>◯ 11-50<br>◯ 51-100<br>◯ 101-500<br>◯ >500
15	How many of these cases are patients who have no standard therapeutic options?	2<br>4<br>3<br>4<br>3	◯ 100%<br>◯ 75%<br>◯ 50%<br>◯ 25%<br>◯ 10% or less
16	Clinical cases are presented by:	6<br><br><br><br>5<br><br>4<br>0<br><br>0<br>1	◯ The medical oncologist in charge of the patient with no prior discussion by the Oncology Multidisciplinary Groups (OMG) or a Disease management Team (DMT)<br>◯ The medical oncologist following prior GOM/DMT discussion<br>◯ Any other clinician<br>◯ Any MTB professional (e.g. a pathologist, radiotherapist etc)<br>◯ On patient's request<br>◯ Other (specify)
	Specify 1 All of the above		__________________________

18	Are any professional(s) appointed who will bear responsibilities for case discussion and clinical management until an MTB final recommendation is made?	3	◯ Oncologist
		0	◯ Biologist/Molecular Pathologist
		12	◯ Both
		0	◯ No specific appointment
19	Do you have an institutional MTB website?	13	◯ No
		2	◯ Yes, only accessible by the intranet to MTB members and professionals working at our institution
		1	◯ Yes, accessible via intranet, but also open to external professionals who may present their clinical cases
		0	◯ Yes, intranet, external professionals, and patients who may access to ask for MTB consideration
20	Do you have an institutional e-mail address?	5	◯ No
		5	◯ Yes, but only for in-house MTB managing
		4	◯ Yes, for medical personnel only, including doctors from other institutions
		2	◯ Yes, available on the institutional website and accessible to the patients to ask for info
21	What tumor is most often referred to your MTB? (you may thick more than one box)	10	☐ Lung
		4	☐ Mammary
		4	☐ Brain
		0	☐ Melanoma
		3	☐ Colorectal
		1	☐ Urogenital
		0	☐ Hematological
		5	☐ Rare tumors
		7	☐ All tumors are eligible

22	Do you use a case annotation/information retrieval solution?	2	◯ Yes, commercially available (1), e.g. REDcap
		3	◯ Yes, harmonized with hospital electronic records (2)
		0	◯ Yes, dedicated and custom-designed for your institutional workflow
		1	◯ A combination of 1 and 2
		1	◯ A combination of 1 and 3
		3	◯ A combination of 2 and 3
		6	◯ No, no platform available
23	If you have a bioinformatic solution, which functions are integrated? (you may thick one or more boxes)	6	☐ MTB meeting (calling)
		7	☐ MTB meeting (briefing)
		5	☐ Patient identification, coding, pseudo-anonymization, data registration, etc
		6	☐ Annotation of clinical history
		4	☐ Type of molecular profiling performed
		7	☐ Molecular profiling results
		7	☐ Clinical Follow-up
		6	☐ Querable records, biostatistics capability
		6	☐ Filing MTB report
24	Do you collect an informed consent authorizing the collection of clinical specimens and case discussion?	5	◯ Yes
		8	◯ No, patients sign a generic consent to biobanking and testing (this is offered to all of our institutional patients)
		3	◯ Other (specify)
	Specify		2 To be defined<br>1 No informed consent obtained
25	Molecular profiling is carried out on the following specimens (more than one box may be ticked)	17	☐ Metastatic tumor
		17	☐ Primary tumor
		14	☐ Blood (liquid biopsy)
26	How are tumors profiled?	9	◯ In-house
		0	◯ Outsourcing (in collaboration with another public institution)
		0	◯ Outsourcing (private provider, for-profit)
		0	◯ Outsorcing (multi-national vendor e.g. F-One)
		7	◯ Mixed >50% in-house
		1	◯ Mixed >50% outsourced non-profit
		0	◯ Mixed >50% outsourced, for-profit

27	Which sequencing tools are available to your MTB?	12 ◯ Targeted NGS (panels)<br>1 ◯ Whole exome sequencing<br>0 ◯ Whole genome sequencing<br>1 ◯ RNAseq<br>0 ◯ None<br>3 ◯ Other (specify)
	Specify 1 All of the above	____________________________________
	Do you make use of liquid biopsy?	15 ◯ Yes<br>2 ◯ No
28	If you use liquid biopsy, which analytes do you look for? (you may tick more than one box)	2 ☐ CTC<br>2 ☐ Exosomes<br>15 ☐ ctDNA<br>0 ☐ Other (specify)
	Specify	____________________________________
30	Which NGS platforms are available to your MTB? (you may tick more than one box)	13 ☐ ThermoFisher<br>2 ☐ Qiagen<br>13 ☐ Illumina<br>1 ☐ Other (specify)
	Specify 1 Archer	____________________________________

	Besides NGS do you adopt any other approach to assign off-label therapy? (you may tick more than one box)	14<br>7<br>2	☐ PCR assays<br>☐ Cytogenetics<br>☐ Other (specify)
	Specify		1 FISH<br>1 Sanger, NGS, FISH
31	What kind of NGS targeted panels are available to your MTB?	4<br>2<br>4<br>4<br>3	◯ A single (or a series of) tumor-agnostic panel(s), e.g. applicable independently of tumor histotype<br>◯ Tumor-specific panels<br>◯ Both solutions applied approximately to the same extent<br>◯ Both, but tumor-agnostic solutions most utilized<br>◯ Both, but tumor-specific solutions most utilized
32	How many genes in the NGS panels used by your MTB? (you may tick more than one box)	3<br>3<br>8<br>0<br>6	☐ >10 genes<br>☐ >20 genes<br>☐ >50 genes<br>☐ >100 genes<br>☐ >200 genes
33	How many genes in the NGS panels used by your MTB for liquid biopsy? (you may tick more than one box)	4<br>5<br>4<br>2<br>3	☐ >10 genes<br>☐ >20 genes<br>☐ >50 genes<br>☐ >100 genes<br>☐ > 200 genes
34	How do you score actionable genomic alterations? (you may tick more than one box)	11<br>11<br>4<br>2	☐ ESCAT-ESMO<br>☐ OncoKB<br>☐ AMP-ASCO-CAP<br>☐ Other (specify)
	Specify		1 Literature search<br>1 Bioinformatic analysis

35	What is the molecular diagnosis turnaround time?	11<br>6	◯ ≤15 days<br>◯ >15 days
36	Do you consider liquid biopsy reliable to assign therapy when the molecular alteration is not seen in tissues?	12<br>3	◯ Yes<br>◯ No
37	Do you have an MTB reporting format distinct from standard molecular reports?	11<br>4	◯ Yes<br>◯ No
38	If yes, does the MTB simply recommend a specific therapy at a specific actionable level, or does it provide a complex case discussion?	5<br>6	◯ Simple report<br>◯ Complex report
39	Who communicates testing results to the patient?	16<br>0	◯ Medical Oncologist<br>◯ The MTB
40	If an off-label drug is recommended and nominal/compassionate use is unavailable, how is the drug payment secured?	5<br>2<br>6<br>4	◯ National Health System (AIFA 5% Fund)<br>◯ Directly Supported by the Medical Oncology Unit<br>◯ Enrolment in Clinical Trials<br>◯ Other (specify)
	Specify 4 All of the above		__________________________
41	Is your center partecipating in National or International target therapy, genome-driven trials?	6<br>11<br>0	◯ Yes, National<br>◯ Yes, International<br>◯ No

ACC-MTB network supplementary survey

Welcome! Please fill out the form by answering all questions.
Thank you

ACC-MTB network supplementary survey

This supplementary questionnaire integrates the original MTB-ACC survey. There are no 'right' or 'wrong' responses. Results will be anonymised before sharing. The identity of responders will be encrypted and by no means specific responses will be associated to the identity of the ACC respondent Member

---

Does a large NGS panel (e.g. 500 genes) involve a delay in diagnostic turnaround time?

- 4 ○ Yes
- 6 ○ No
- 1 ○ No (no 500-gene panels available, and/or no multiple panel comparison possible)

To which extent (days)?

- 1 ○ 5
- 2 ○ 15
- 1 ○ >15

Do Whole Exome Sequencing and other untargeted genomic profiling approaches involve a diagnostic delay?

- 4 ○ Yes
- 1 ○ No
- 6 ○ No Whole Exome Sequencing performed and/or no comparisons possible

To which extent (days)?

- 0 ○ 5
- 2 ○ 15
- 2 ○ >15

Your MTB recommends non-standard (off-label) therapy: out of 100 eligible patients how many will actually receive the selected treatment?

- 7 ○ 0-20%
- 3 ○ 21-50%
- 0 ○ 51-80%
- 1 ○ >80%

Which is the most frequent cause preventing the administration of MTB-recommended off-label therapy?

- 7 ○ Drug unavailable
- 0 ○ The medical Oncologist decides not to not apply therapy
- 0 ○ Patient refuses to be treated
- 4 ○ Deteriorated general conditions prevent therapy
- 0 ○ Other

Specify other

____

Whichever the the most frequent cause preventing off-label therapy, which is its prevalence?

- 1 ○ >90% of cases
- 3 ○ >70% of cases
- 2 ○ >50% of cases
- 4 ○ >20% of cases

When patients treated off-label are EXCLUSIVELY considered, how is treatment applied? (you may tick more than one box)

- 9 ☐ Clinical trial
- 8 ☐ compassionate use
- 7 ☐ AIFA 5%
- 1 ☐ Other

Indicate the percent of patients treated in the context of a clinical trial	3 ◯ 0-25%<br>3 ◯ 26-50%<br>1 ◯ 51-75%<br>2 ◯ 76-100%
Indicate how many patients treated off-label receive treatment through compassionate	5 ◯ 0-25%<br>3 ◯ 26-50%<br>0 ◯ 51-75%<br>0 ◯ 76-100%
Indicate how many patients treated off-label receive treatment through AIFA 5%	6 ◯ 0-25%<br>1 ◯ 26-50%<br>0 ◯ 51-75%<br>0 ◯ 76-100%
Indicate how many patients treated off-label receive treatment through other economical resources	1 ◯ 0-25%<br>0 ◯ 26-50%<br>0 ◯ 51-75%<br>0 ◯ 76-100%
Specify other economical resources<br>1 Foundations and Charities	__________________________________
If a tumor displays >1 actionable alteration, how do you prioritize treatment? (more than one box may be ticked)	4 ☐ Specific genomic alterations are often prioritized over multigene signatures (e.g. KRASG12C>mutation burden)<br>0 ☐ The opposite<br>1 ☐ A combination treatment is seriously considered in specific cases only<br>9 ☐ The choice depends on clinical and pharmacogenomic considerations, and the specific clinical case. We have no guidelines, we search literature and decide case by case<br>0 ☐ It is a rare occurrence, or it was never observed by us
